# Road traffic injury among young people in Vietnam: evidence from two rounds of national adolescent health surveys, 2004–2009

**DOI:** 10.3402/gha.v6i0.18757

**Published:** 2013-01-17

**Authors:** Linh Cu Le, Robert W. Blum

**Affiliations:** 1 Department of Demography, Hanoi School of Public Health, Hanoi, Vietnam; 2 Department of Population and Family Health Science, Johns Hopkins Bloomberg School of Public Health, Baltimore, MD, USA

**Keywords:** road traffic injury, adolescent, risk and protective factors, Vietnam

## Abstract

**Background:**

Based on previous data, road traffic injury (RTI) was a leading cause of non-fatal injury in all-age groups in Vietnam, and among the top causes of injury in children and adolescents. Specific analysis on RTIs in young people, however, has yet to be fully investigated. Using the results of two surveys in 2004 and 2009, the present study aims to describe the current situation of non-fatal, unintentional RTIs among Vietnamese youths. In addition, it explores RTI-related risk and protective factors.

**Methods:**

This study utilized the nationally representative Survey Assessment of Vietnamese Youth 2009 (SAVY2) of 10,044 youths aged 14 to 25 from all 63 provinces in Vietnam. The indicators were compared with data from SAVY1 in 2004 of 7,584 youths. Bivariate and multivariable statistical techniques were applied.

**Results:**

Overall, 75% of youths used a motorcycle in SAVY2 compared with 54.2% in SAVY1. Of the SAVY2 sample, the proportion that had experienced an RTI was 10.6% vs. 14.1% in SAVY1. While the proportion of RTIs for both sexes decreased, the decline was greater for males (11.9% vs. 17.8% in SAVY1) than in females (9.2% vs. 10.4%). The proportion of rural youths aged 22–25 who experienced an RTI increased slightly in the 5 years between the two study intervals. The percentage of youths reporting frequent helmet use increased significantly from 26.2% in SAVY1 to 73.6% in SAVY2. Factors related to the likelihood of ever having experienced an RTI included: older age, male, ever being drunk, and ever riding motorcycles after drinking.

**Conclusion:**

While improvements in RTIs appear to have occurred between 2004 and 2009, more attention should be paid, particularly, in maintenance and supervision of law enforcement to helmet use and drunk driving.

It is estimated that road traffic crashes kill about 260,000 children and adolescents worldwide under the age of 18 every year. Recognizing the huge burden of such loss, the United Nations launched an initiative in 2011 called a ‘Decade of Action for Road Safety’ with the aim of stabilizing and then reducing global road deaths by 2020 (1). With dramatic economic growth and the improvement of living standards, Vietnam has experienced a rapidly growing number of motor vehicles on its roads in recent years; however, motorcycles still represent the main mode of transport with 26,869,025 motorcycles registered in 2010 (2). The pattern of unintentional injury of adolescents and young adults in Vietnam had previously been periodically studied in several surveys. The first was the Vietnam Multi-center Injury Survey (VMIS) conducted in 2001. That study showed that among young people under 20 years of age in Vietnam, injury accounted for 70% of the burden of disease using the years of potential life lost (YPLL) measurement, compared to 17% due to chronic diseases and only 13% due to communicable diseases (3). Among all injuries, traffic-related fatal injury is the leading cause of death in Vietnam (4). Road traffic injuries (RTI) is also a third leading cause of non-fatal injury in the under 20 age group. Children and adolescents in Vietnam experienced an alarmingly high rate of non-fatal injury with 4,818 episodes per 100,000 population, with a mortality rate of 26.7 per 100,000 inhabitants per year.

**Policy implications and recommendations**

- Injury control and prevention efforts will improve significantly if more attention is directed at the maintenance and supervision of law enforcement on helmet use and drunk driving control measures in the promotion of traffic safety behaviors for young people.
- As injury patterns now show that females are becoming more vulnerable to road-traffic-related injury, no adolescent health policy should overlook this issue in its injury control design.

It is also worth mentioning that from 2001 to 2003 the Ministry of Transport of Vietnam had issued several guidelines stipulating types of roads on which helmet use is mandatory when motorcycling. The circular was then complemented by Resolution No. 13/2002/NQ-CP on traffic safety (5, 6). The government's regulations have forced, to some extent, motorcycle riders to use helmets in the inter-provincial roads and national highways. These regulations, however, did not order helmet use in urban areas. As a consequence, rural residents have tended to produce better adherence than their urban counterparts.

In 2004, the Ministry of Health undertook the first ever comprehensive national survey of youth in Vietnam, covering a sample ranging from 14 to 25 year olds across the country which became known as the Survey Assessment of Vietnamese Youth (SAVY1). SAVY1 data showed that injury most likely takes place on the highway and street, accounting for 59.8% of all injuries (higher in urban: 68.2% vs. 55.8% in rural settings) followed by injury in the home and at the work place (16.7% each). The key findings from SAVY1 suggested that RTI accounted for a vast majority of all injury types and motorcycle injuries predominated. Overall, 54.2% of youths had used a motorcycle as a driver or passenger (7). In 2004, when SAVY1 data were collected, there was no compulsory helmet use requirement in metropolitan areas. SAVY1 data made it clear that legislation was central to helmet use: 51.9% of youths indicated that the law influences their decision compared with 37% who said injury avoidance was a major motivation for helmet use, and only 4% who said road traffic safety education was key. School-based education programs and free helmets were found to have very little influence overall (8).

Recognizing the burden of injury, in June 2007, the Vietnamese Government decreed that by the end of that calendar year all motorcycle drivers and passengers had to wear a helmet on all roads (9). Several studies have been conducted to investigate the impact of this resolution. Some were conducted before and some after the decree in order to assess the changes. Pervin et al. (10) found that the frequency of helmet use in the four study locations ranged from 90 to 99% among adults, but only 15–53% among children 7 years of age or younger, and from 38 to 53% among children aged 7–14. It has been said that legislation to penalize adults whose children do not wear motorcycle helmets has been proposed in Vietnam. However, ongoing advocacy and social marketing efforts should be improved to disseminate information about the safety benefits of helmets to combat erroneous public perceptions (10).

To follow-up results from SAVY1, SAVY2 was conducted 5 years later in 2009. The major findings on SAVY2 have since been reported (11). However, no in-depth analysis has been performed specifically for motorcycle riding, helmet use and prevalence of RTI by demographic characteristics, particularly the risk factors associated with RTI among Vietnamese youths. This paper (1) compares the prevalence of motorcycling, helmet use and prevalence of RTI among youths in Vietnam in 2004 and again in 2009 and (2) determines the risk and protective factors associated with RTI among Vietnamese youths.

## Methods

### Study sites and sampling strategy

In 2004, the Survey Assessment of Vietnamese Youth (SAVY1) was conducted by the Ministry of Health of Vietnam. The study involved a cluster-sample of 7,584 youths aged 14 to 25 years from 42 provinces (out of 61 provinces) across the country. SAVY2 was then conducted in 2009 as the follow-up. A household sample was used based on the Vietnam Living Standard Survey (VNLSS 2008) sampling frame. SAVY2 was also a multi-stage cluster sample covering all 63 provinces throughout Vietnam. Data collection was from mid-May to the end of June 2009. Similar to SAVY1, young people were invited to come to a central location to complete both an interview and a self-administered survey. Of those invited to participate in SAVY2, 86% agreed, resulting in a final total of 10,044 young people.

### Instruments

The SAVY2 instrument was designed to assure comparability with SAVY1 questions and covered a wide range of topics: demographics; education; work; puberty; dating and relationships; reproductive health; HIV/AIDS; injury, illness, and physical health; knowledge/attitudes/beliefs regarding a range of issues; violence; mental health; and mass media and aspirations. The questionnaire was anonymous and contained both interviewer-led and self-completed questions (the latter for more sensitive questions).

Ten specific questions related to unintentional injury, particularly RTI, included 1) Have you had any accident or injury during the last 12 months that required medical treatment? 2) Where did you have that accident/injury? 3) Have you ever ridden a motorcycle? 4) Do you wear a helmet when driving or as a passenger on a motorcycle? 5) In the last 6 months, did you ever travel on a motorcycle without a helmet? 6) Give the main reason that may make you wear a helmet regularly? 7) Have you ever had a road traffic accident because of which you had to take at least 1 day off for treatment? 8) In the last 12 months, did you have a road traffic accident because of which you had to take at least 1 day off for treatment? 9) Have you ever ridden a motorcycle/car after drinking alcohol? 10) Have you ever traveled with a driver who had drunk alcohol? Additionally, respondents were also asked to report their alcohol intake.

### Data analysis

The present analysis included socio-demographic characteristics of the respondents (sex, age, ethnic group, place of residence, household economic status), their alcohol and motorbike riding behaviors, and the RTI-related variables of the SAVY2 dataset. The results of SAVY2 were then compared to the findings in SAVY1.

SPSS package version 16.0 was used for data management and manipulation. The individual record in the dataset was weighted to adjust for complex sampling design, making the results nationally representative. Data were first analyzed using univariate and bivariate statistical techniques (with Chi-square statistics). Then, to understand associated factors, multivariable analyses (using binary logistic regression) were performed to identify the strongest predictors of RTI in these young people. In two regression models, a selected set of independent variables were explored: age group (three categories: 14–17, 18–21, 22–25 years old); sex (males vs. females); area of residence (urban vs. rural); ever been drunk (yes/no); ever ridden a motorbike after drinking alcohol; and geographical regions (Red River Delta, North East, North West, North Central, Central Coast, Central Highland, South East, and Mekong River Delta). Of the geographical regions, the most disadvantaged regions are the two mountainous areas in the north (North East and North West) and Central Highland in the south. In contrast, the most urbanized and economically developed regions are Red River Delta (where the capital city of Hanoi is located) and the South East (where the largest and most prosperous city, Ho Chi Minh City, is located).

These multiple logistic regression models were performed in enter mode. The first model predicted the likelihood that the respondent has ever suffered an RTI as the dependent (outcome) variable. The second model analyzed the likelihood that the respondent suffered an RTI in the last 12 months prior to the survey. A chi-square goodness-of-fit Hosmer and Lemeshow test was performed, and an adjusted odds ratio (OR) and 95% confidence interval of OR were reported and compared with the crude OR.

## Results

### Sample characteristics and overall injury context

Approximately 75% of youths in the SAVY2 sample lived with their biological parents. Among 10,044 respondents, males accounted for 51% (vs. 49% females), and age groups 14–17, 18–21 and 22–25 accounted for 48%, 29% and 23%, respectively. The majority of the sample was unmarried (83%) and, consistent with the population as a whole, 75% resided in rural areas. Similar to SAVY1, SAVY2 asked young people whether they had an injury requiring medical treatment in the previous 12 months; 6.6% of respondents answered *yes* (significantly lower than the 7.4% in SAVY1, *p*<0.05). Males had a higher prevalence of injury than females (8.0% vs. 5.2%, *p*<0.05), but this difference was less than that found in SAVY1 (11.0% male vs. 3.7% female). As in the previous survey, SAVY2 data show that injury most likely takes place on the highway and street. SAVY2 findings show that the gap between road and home or work injury is much greater than previously reported. Specifically, SAVY2 showed that road injury accounted for 73.3% of all injuries (compared to only 59.8% in SAVY1) and was more common among females than males both in urban (75.3% male vs. 85.9% urban female) and rural settings (67.6% male vs. 76.4% rural female).

### Motorcycling and helmet use

Overall, 75% of youths used a motorcycle as a driver or passenger increasing from 54.2% in SAVY1 (*p*<0.01). Not surprisingly, motorcycle use increased with age. Likewise, males were more likely to ride or drive motorcycles than females, and the Kinh – the majority population – was more likely to ride motorcycles than were ethnic minorities. Additionally, SAVY2 showed an increase of motorcycle use in urban areas among both females and males to where now it is nearly universal (see Table 1). Though not quite as universal, in rural areas dramatic increases were seen as well in the years between 2004 and 2009. For example, among rural females the increase went from 45.5% motorcycle use in 2004 to 80.5% 5 years later). Likewise, over the 5-year interval the gap between rural and urban motorcycle use narrowed.

**Table 1:** Percent of ever used motorcycle by gender and location^a^

Age groups	SAVY1				SAVY2			
	
	14–17	18–21	22–25	14–25	14–17	18–21	22–25	14–25
Urban male	53.9	89.2	95.8	77.4	66.9	95.8	99.0	83.7
Urban female	43.5	72.8	86.8	64.2	50.0	94.4	94.2	74.2
Rural male	40.6	74.3	80.2	59.5	68.9	93.4	94.7	82.0
Rural female	25.6	50.6	45.8	38.4	50.3	79.1	80.5	65.0
All groups	36.6	67.1	70.5	54.2	59.3	88.7	90.4	75.0

a All percents in SAVY2 were significantly higher than those in similar age group, sex, and location found in SAVY1 (*p*<0.05).

Turning to helmet use we see a dramatic increase when comparing SAVY1 and 2; and that increase is seen among all age groups. Overall the increase was from 26.2% reported helmet use in SAVY1 to 73.6% helmet use 5 years later. For the oldest age group (22–25 year olds), the increase was from 35.9 to 82.7%.

**Fig. 1:**
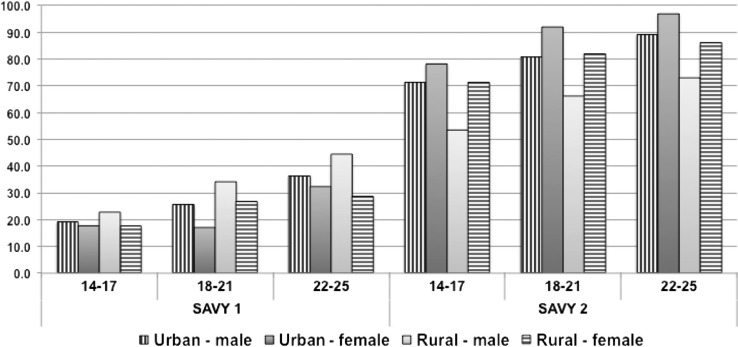
Percent of always wearing helmet by gender and location.

Helmet use is analyzed by age group and geography, and it is clear that the pattern of use has changed. Specifically, in SAVY1, 22–25-year-old rural males accounted for the highest proportion of helmet use (44.5%), followed by same age urban males (36.4%). This pattern has now changed. In 2009, the helmet use proportion for rural males was more than twice that seen in SAVY1 (62.9% vs. 30.9%), and for rural females the increase was three-fold (78.9% vs. 23.1% in SAVY1). Among urban youth, the changes were even more dramatic. In 2009, 79.8% of urban males reported helmet use compared with 25.7% 5 years earlier. And for females the rate climbed to 89.3% from 21.4% in SAVY1. Urban females are now the group with highest proportion of helmet use – a dramatic shift in 5 years (Fig. 1).

Beyond urban and rural changes, there is also another shift in helmet use over the years before and after compulsory helmet use was instituted. While in SAVY1 helmet use was disproportionately higher in the northern provinces, by 2009 that had shifted to the southern regions of the country (e.g. 94.4% in the Mekong River Delta region). This is of significance since the population and motorcycle density is greater in the south (see Table 2).

**Table 2:** Percent of always wearing helmet while using motorcycle by age group and region^a^

Age groups	SAVY1				SAVY2			
	
	14–17	18–21	22–25	14–25	14–17	18–21	22–25	14–25
Red River Delta (highly urbanized)	19.9	29.8	41.6	28.0	37.6	55.6	70.2	53.2
North East (mountainous region)	34.9	40.4	42.3	38.5	49.5	67.8	78	64.3
North West (mountainous region)	20.3	26.1	26.1	23.2	38.4	57.5	56.9	50.4
North Central	27.4	34.0	41.1	31.8	63.5	76.3	78.2	71.2
Central Coast	16.4	23.5	34.3	22.9	67.1	80.3	87.4	76.6
Central Highland (mountainous region)	11.5	28.1	31.9	20.9	67.7	74.5	77.5	71.8
Southeast (highly urbanized)	11.2	17.6	26.9	17.2	78.5	87.1	93.5	86
Mekong River Delta	15.9	25.6	32.9	23.3	92.5	94.5	96.7	94.4
All groups	19.9	28.2	35.9	26.2	63.9	76.8	82.7	73.6

a All percents in SAVY2 were significantly higher than those in similar age group, sex, and location found in SAVY1 (*p*<0.05).

There is another interesting finding when frequency of helmet use is compared with reports of ever having been fined for non-helmet use. While the majority of adolescents and youths indicate that they do not use helmets all the time, those who live in the south – where there is a greater population density – report more consistent use. Also those living in the south report a greater likelihood than their northern peers of being fined for non-helmet use in the previous 12 months (see Table 3).

**Table 3:** Multiple logistic regression models to predict the likelihood of having road traffic injury, SAVY2 (2009)

Independent variables	Model 1: Life time experience of RTI (*N*=10,034)^a^				Model 2: RTI in the last 12 months (*N*=10,021)^b^			
	
	Crude OR	95% CI of crude OR	Adjusted OR	95% CI of adjusted OR	Crude OR	95% CI of crude OR	Adjusted OR	95% CI of adjusted OR
Gender								
Male	1.335	1.175–1.518	1.297	1.101–1.529	1.489	1.218–1.821	1.442	1.112–1.871
Female*	1		1		1		1	
								
Age groups								
22–25	2.869	2.444–3.368	1.905	1.594–2.277	2.354	1.827–3.034	1.508	1.165–1.953
18–21	2.215	1.892–2.594	1.609	1.359–1.905	2.305	1.813–2.933	1.377	1.038–1.827
14–17*	1		1		1		1	
								
Area								
Urban	1.536	1.339–1.763	1.241	1.063–1.448	1.203	0.965–1.498	1.032	0.809–1.317
Rural*	1		1		1		1	
								
Had ever been drunk								
Yes	2.486	2.186–2.427	1.723	1.456–2.040	3.056	2.500–3.737	2.137	1.643–2.779
No*	1		1		1		1	
								
Had ever ridden motorbike after drinking								
Yes	3.022	2.641–3.457	1.932	1.614–2.312	3.464	2.834–4.235	2.271	1.729–2.982
No*	1		1		1		1	
								
Region								
Red River Delta	0.901	0.731–1.111	0.966	0.771–1.209	0.829	0.604–1.139	0.881	0.627–1.239
North East	0.909	0.715–1.154	0.849	0.662–1.088	0.829	0.575–1.195	0.723	0.496–1.056
North West	0.690	0.452–1.053	0.692	0.448–1.069	0.876	0.492–1.559	0.784	0.432–1.424
North Central	0.733	0.569–0.943	0.862	0.664–1.118	0.537	0.353–0.817	0.613	0.399–1.118
Central Coast	1.324	1.026–1.708	1.266	0.972–1.650	1.210	0.825–1.774	1.103	0.742–1.638
Central Highland	1.067	0.803–1.419	1.060	0.790–1.422	1.191	0.793–1.787	1.099	0.724–1.670
South East	1.735	1.423–2.116	1.514	1.229–1.866	1.300	0.957–1.766	1.158	1.842–1.594
Mekong River Delta*	1		1		1		1	

* Reference category.

a Goodness-of-fit Hosmer & Lemeshow test χ^2^=4.533; df = 8; *p*=0.806.

b Goodness-of-fit Hosmer & Lemeshow test χ^2^=8.204; df = 8; *p*=0.414.

### Road traffic injury and drinking behaviors

**Fig. 2:**
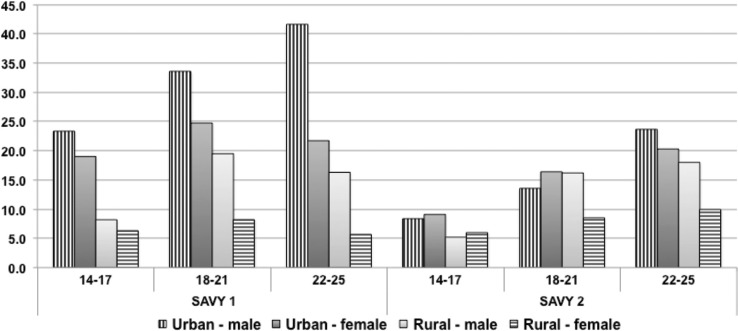
Percent ever had traffic accidents by gender, age group, and location.

The SAVY1 questionnaire did not provide specific questions for identifying RTI prevalence and incidence rates. Instead, questions only asked lifetime traffic injury. These questions were revised in SAVY2. Regarding RTI lifetime injury there is a significant decline over the 5-year interval between SAVY 1 and 2 (10.6% vs. 14.1%, *p*<0.01). Not surprisingly, the figures in both sexes decreased but the gap between males and females in SAVY2 narrowed as well. This decline is reflected in all regions of the country, for all ages and for both males and females (see Fig. 2).

**Fig. 3:**
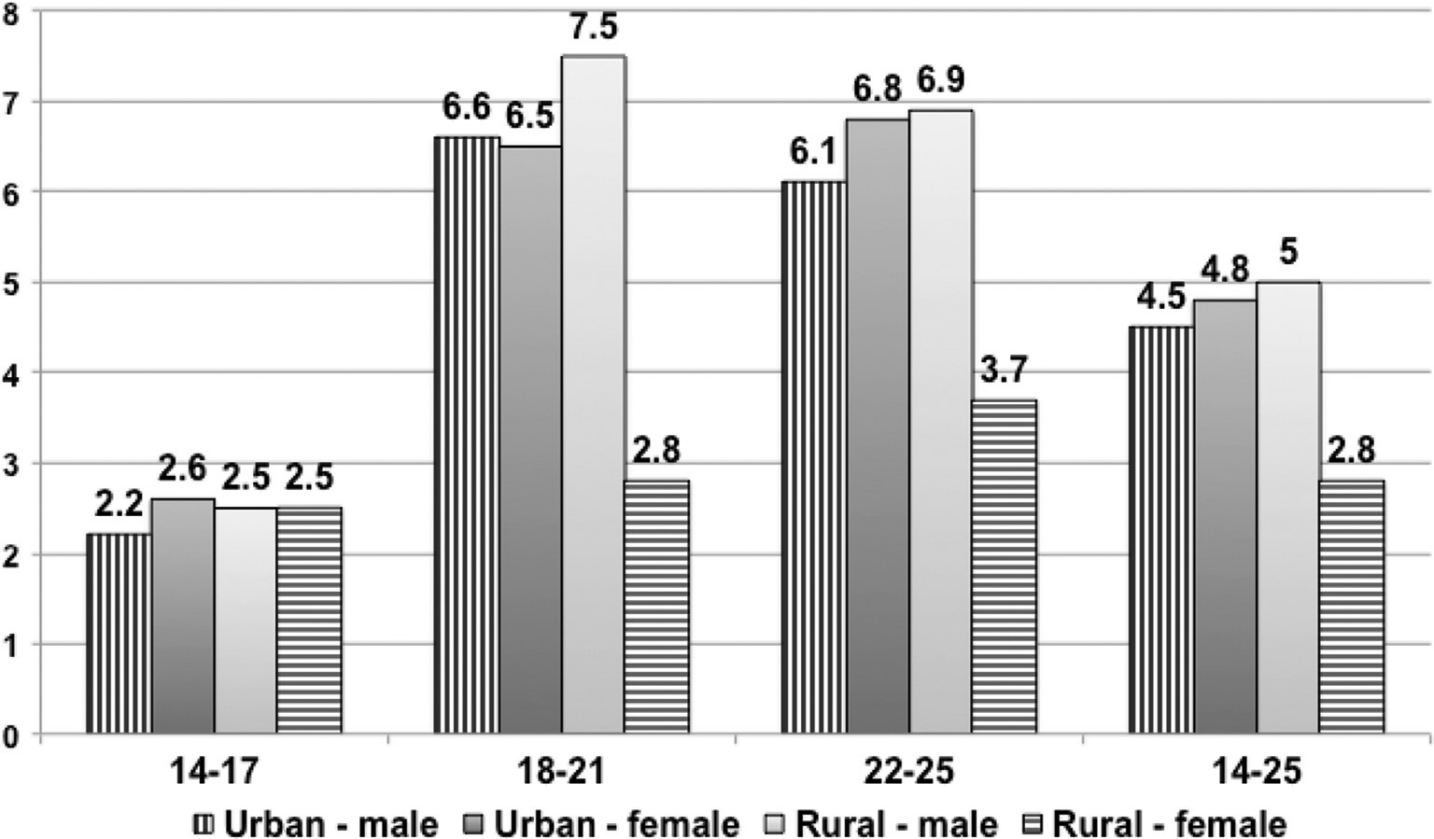
Percent ever had traffic injury in the last 12 months by gender, age group, and location, SAVY2 (2009).

In general, 38.8% of those who ever had an RTI reported it occurred in the previous 12 months. The overall rate was 4.1%; recent injury was highest for males, youths from middle-income families, and those between 18 and 21 years of age. However, there was little ethnic difference seen among those who experienced an RTI in the previous year (Fig. 3).

Drinking and driving is the great concern in any RTI program. SAVY2 data showed that alcohol use is quite common in young people; about 80% of males and 36.5% of females have drunk a glass of beer/liquor. Among those who have tried alcohol, a third report that they have ever ridden or driven a motorbike after drinking. The age-specific proportion of youths riding a motorcycle after drinking alcohol rose from 19% among the youngest group of males to 68.1% among the oldest. For females it was much lower (4.1% among those 14–17 years and 12.7%, among 22–25 year olds). This experience was more common as the economic status of the young people increased. Drinking and driving appears to be more common in urban settings (35.8% vs. 31.6% in rural areas, *p*<0.01). Equally worrisome is the practice of riding with a drink driver, which nearly two thirds of males (6.12%) and nearly half of females (42.9%) admit having done.

### Factors associated with RTI

Multivariable analyses were performed to identify the factors associated with ever having experienced an RTI both over one's lifetime and in the previous 12 months. The two models summarized in Table 3, consistently showed the following risk factors: male, urban, older, ever been drunk, ever ridden motorcycle after drinking, and those who were from the South East region of Vietnam (Model 1) and in the North Central region (Model 2). Specifically, regarding lifetime experience of RTI, youths in the two older age groups (18–21 and 22–25) were found, respectively, at 1.6 and 1.9 times higher risk for RTIs than those in the 14–17 age group. Men were a 1.3 times higher risk than women. Those who lived in urban settings were at 20% more likely to experience an RTI. Youths who reported ‘ever been drunk’ had an RTI risk almost 1.7 times to that of peers who reported to never having been drunk. Importantly, those who acknowledged having ridden a motorbike after drinking were almost two times more likely to have had an RTI. Regarding regional variations, young people in the South East region (which is the most urbanized and economically developed region) were at 1.5 times higher risk of RTI than their counterparts in the Mekong River delta. A similar pattern was found in Model 2 for the likelihood of an RTI in the last 12 months, except that urban/rural settings were not significantly different and the North Central region reported a higher risk.

## Discussion

### Motorcycling and helmet use behaviors

With the rapid pace of economic development and urbanization over the 5-year period between 2004 and 2009, young people use motorcycles more frequently today, and the gaps between rural and urban and between males and females have significantly decreased. In 2009, there was no significant difference in the proportion of motorcycle use between urban and rural males and only a 9% difference between urban and rural females compared with more than a 20% difference only 5 years before. Clearly, this relates to the rapid urbanization as well as the improvement of living standards and income in rural areas. On the other hand, this also helps explain the higher risk of traffic-related injury in youth, especially among young urban males. Given that this is cross-sectional data, it is not possible to determine causality; however, there is a strong possibility that the imposition of a helmet law was what accounted for most of the uptake in use. This conclusion is reinforced by the finding from SAVY2 that, more than anything else, the existence of a law was seen by adolescents and youths to be the most influential factor on their behavior. Likewise, the data suggest that the enforcement of the law (as measured by the likelihood of being fined for infractions) may influence use, thereby explaining the regional differences and the highest use rate in the south where fines were also most common. Only future research will be able to determine causal pathways.

### The pattern of RTI, alcohol drinking, and other related factors

The percentage of those who have ever experienced an RTI when comparing two rounds of SAVY showed improvement. Overall, the figures in urban settings declined in SAVY2. In contrast, the situation in rural areas showed little change. In fact, the proportion of RTIs in both rural males and females aged 22–25 was slightly higher compared with SAVY1. This pattern probably corresponds to greater motorcycle use in rural regions in 2009 when compared with 5 years earlier.

Ever having been drunk is a significantly associated risk for traffic-related injury (12). Given the fact that adolescent alcohol use in Vietnam is high compared to many countries in developing world (13), it is important to analyze the drink driving issue. In our present analyses, we see a strong association between ever having been drunk and RTI. Again, the data do not allow us to conclude a causal pathway. It may be that the association is because of drinking and driving or it may be that the factors that are associated with alcohol use in adolescence are parallel factors to those that predispose to RTI. Only future analyses will allow for clarification. What we do know from SAVY2 is that there is an increasing trend for youths to have had ridden a motorcycle after drinking. Similarly, 62.1% of males and 42.9% of females admitted that they had ridden with a driver who had consumed alcohol. This alarming finding is comparable to other studies in Vietnam that reported a significant proportion of respondents aged 17 and above (44.9%) were drink and drive (14). Multivariable models in the present analyses confirms the known risk factors (15, 16): males, older age groups, experiencing being drunk and having ridden a motorcycle after drinking, which again highlight the serious issue of drunk driving. These data strongly suggested that interventions aimed at curbing drinking and driving could have a dramatic impact on injury outcomes.

Some limitations of this study should be noted. The cross-sectional nature of both SAVY1 and SAVY2 does not allow us to analyze further the causal relationships among those variables. In addition, drinking alcohol was asked as a ‘life-time experience’ disallowing a time-sequence construction of potentially related behaviors. Additionally, no data were collected to allow for calculation of the amount of alcohol consumption. Thus, it is not possible to know for certain the relationships between the timing of drinking, the amount of alcohol consumed, and driving behavior. Recall may be an additional limitation since SAVY asked about both drinking and RTIs in the past year or ever before. The long recall period may lead to bias. So too, the reporting of both alcohol drinking and helmet use may be subject to over- or under-reporting bias depending on perceived social desirability of respondents. The sampling design of these two rounds of SAVY did not allow us to interview the same individuals or panel of youths over time. Therefore, it is not possible to measure change of behavior for the same individuals over time.

## Conclusion

SAVY2 reported a decreased rate of road-traffic-related injury from 5 years previously. As was true in SAVY1, higher injury rates persist in urban areas and in the more populous south of the country. That having been said, the rates of injury appear to be declining especially among urban males. In contrast, there appears to be little change in rural areas. Importantly, SAVY2 data confirmed that alcohol use is strongly associated with risk of experiencing an RTI.

## Policy implications

Within the framework of the national policy for accident and injury prevention (which is now in the process of revision and updating) injury control and prevention will improve if attention is paid to enforcement of helmet laws as well as those laws that prohibit the use of alcohol prior to driving. Enforcement should be a high priority for reduction of RTI. Additionally, special attention should be paid to adolescent and young adult males where both alcohol use and driving are the highest. However, broader-based campaigns need also to be planned since our data indicate that there is an increase in RTI among adolescent females.
